# Traumatic divergent dislocation of the elbow in the adults

**DOI:** 10.1007/s00264-022-05679-5

**Published:** 2023-01-09

**Authors:** Maoqi Gong, Hanzhou Wang, Xieyuan Jiang, Yang Liu, Junlin Zhou

**Affiliations:** 1 Department of Orthopedic Surgery, Beijing Chaoyang Hospital, Capital Medical University, 8 Gongren Tiyuchang Nanlu, Chaoyang District, Beijing, 100020 People’s Republic of China; 2 Department of Orthopedic Surgery, Beijing Jishuitan Hospital, Beijing, 100035 People’s Republic of China

**Keywords:** Divergent dislocation of the elbow, Trauma, Fracture-dislocation of the forearm, The adults, DASH, Multiple injuries

## Abstract

**Purpose:**

This retrospective study aimed to investigate the clinical outcomes of DDE in adults.

**Methods:**

From September 2010 to March 2020, adult patients with traumatic DDEs admitted to Beijing Chaoyang Hospital and Beijing Jishuitan Hospital were included in this study. Each patient underwent operative or conservative treatment during hospitalization. The clinical and radiological examinations were followed up. The primary outcomes included the Mayo Modified Wrist Score (MMWS), the Mayo Elbow Performance Score (MEPS), the Broberg and Morrey functional index, the Disabilities of the Arm, Shoulder and Hand (DASH) score, and the Visual Analogue Scale (VAS) score that were performed. Post-operative complications and secondary surgery details were also collected.

**Results:**

Of the fourteen patients, clinical and radiographic results were reviewed at a mean of 53.2 months (18 to 110 months) postoperatively. There were 11 men and three women with an average age of 31.5 years (17 to 51 years). At the final follow-up, the average MMWS, MEPS, Broberg and Morrey functional index, and DASH scores were 91.4 points, 93.4 points, 92.6 points, and 10.7 points. The mean VAS at rest and during activities was 0.4 and 1.7 points. Two patients required a secondary procedure due to radial malalignment and elbow contracture, respectively. In addition, two patients were found degeneration.

**Conclusions:**

Within the context of high-energy DDE combined with simultaneous upper limb injuries, our study recommended obtaining the mechanical benefit of the forearm ring with concentric elbow stability. Despite the various and complicated traumatic patterns of DDE, great clinical results could be acquired based on adequate surgical treatments and early rehabilitation training.

## Introduction

Divergent dislocation of the elbow (DDE) is an exceedingly rare elbow dislocation in which the distal humerus is caught between the proximal radius and ulna and then brings about the divergence of the proximal forearm bones [1]. All elbow articulations are involved, including humeroulnar, humeroradial, and proximal radioulnar joints. DDE has been divided into two types: the anteroposterior and the transverse dislocation, most commonly occurring in children.

To our knowledge, the first modern case of traumatic DDE was reported by DeLee in 1981 [1]. Since then, thirty-one cases were identified in reported studies, of which only five clinical literatures investigated this injury in adults [1–28]. Most of these studies mentioned that conservative treatment could acquire great clinical outcomes. However, the eventual results of closed reduction of DDE depend on concomitant fractures and the damage degree. So far, the surgical indication of DDE remains an ongoing debate. It is disastrous that emergency surgeons underestimate this trauma pattern on initial evaluation and subsequent treatment in a delayed timing, which can result in elbow dysfunction and other post-operative complications. Moreover, other pathological data, including elbow joint stability and clinical functional scores associated with DDE, remain a relatively unworked area.

Hence, the purpose of this retrospective study was to report the traumatic DDE in adults and to discuss the present management, clinical outcomes, and functional scores.

## Methods

### Study design and participants

This retrospective cohort study was approved by the Ethics Committee of Beijing Chaoyang Hospital and Beijing Jishuitan Hospital to evaluate the medical records and radiographic outcomes of patients receiving treatments for DDE between September 2010 and March 2020. The Committee waived the requirement for written informed consent because the study was retrospective, did not have any adverse effect on patients’ health, and reported anonymized patient data.

The inclusion criteria consisted of adult patients who had suffered from a DDE. The diagnosis of a DDE was referred to a combination of clinical signs and radiographic evidence: elbow dislocation with separation of proximal radius and ulna in opposite directions. Exclusion criteria consisted of patients with a follow-up of less than 12 months, attendance more than two weeks after the injury, and patients requiring a secondary procedure after failed initial treatment in another hospital as a second-stage or salvage procedure.

### Interventions

**Fig. 1:**
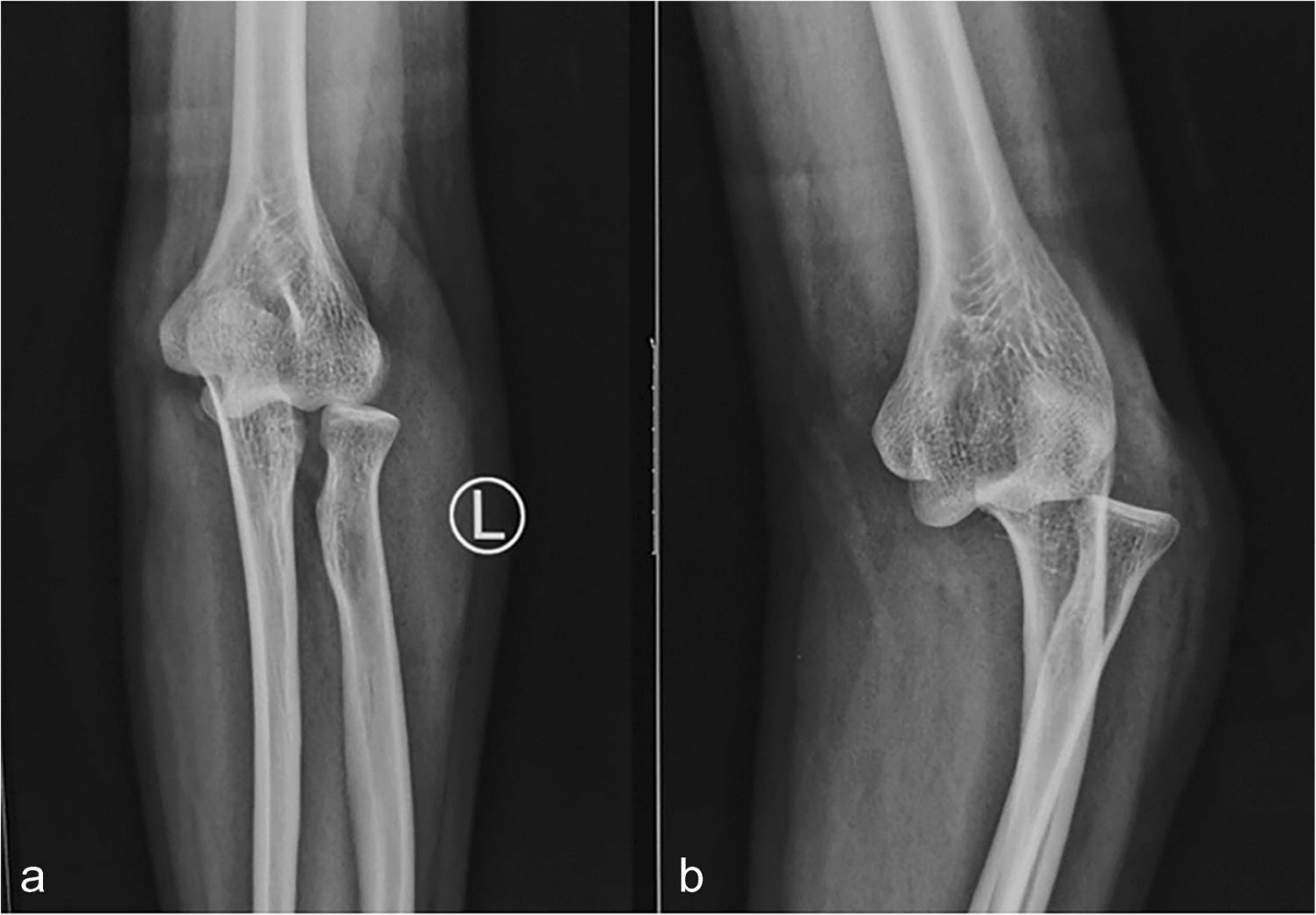
Anteroposterior and lateral X-ray radiographs show an isolated divergent dislocation of the elbow (**a**–**b**)

**Fig. 2:**
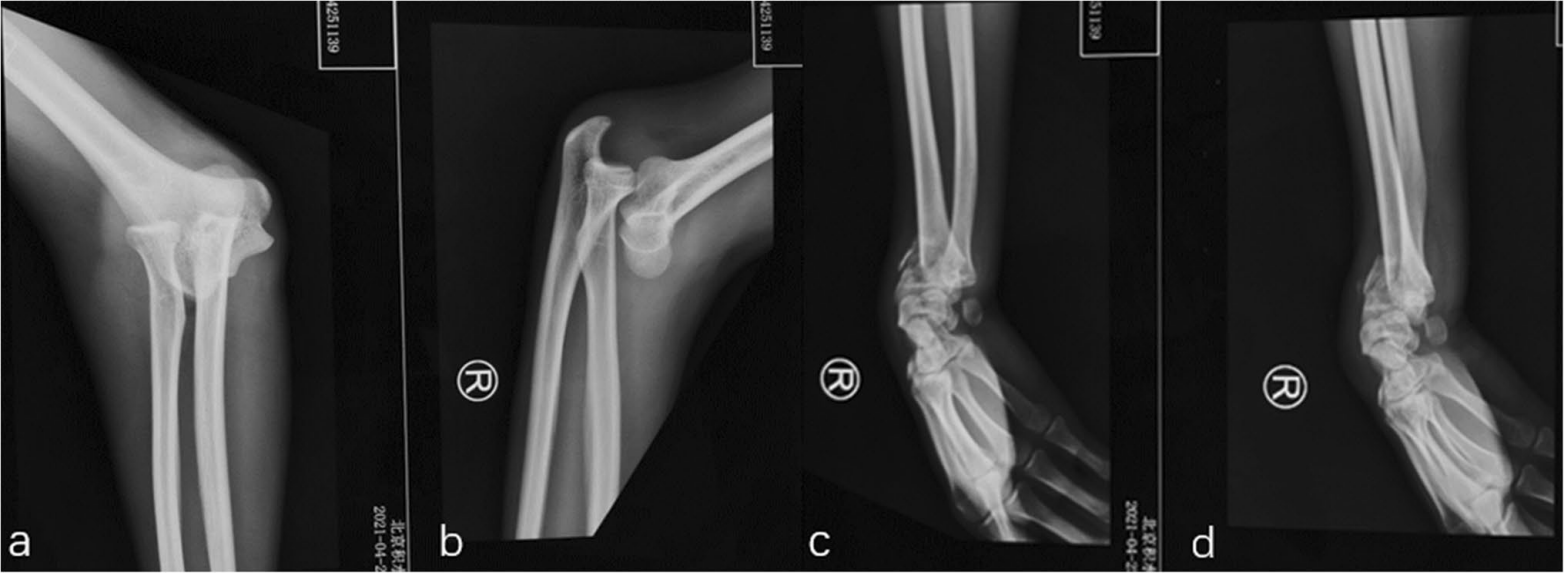
Pre-operative radiographs show a divergent dislocation of the elbow (**a**–**b**) combined with a distal radius fracture (**c**–**d**)

The details of initial and secondary operative procedures are summarized in Table 1. Each patient initially received a closed reduction by distal traction on the forearm with counter traction and compression of the proximal radius and ulna, which has previously been reported [29]. The clinical outcomes were confirmed radiologically. In order to determine the details of concomitant injuries and better perform subsequent treatments, CT scans were performed in twelve cases after a failed closed reduction. The elbow was reduced and obtained stability with a closed reduction in two patients: Figs. 1 and 2 demonstrate an isolated DDE and a DDE combined with a distal radius fracture.

**Table 1:** Characteristics of patients undergoing divergent dislocation of elbows

No	Sex	Age	Side	Causes of injury	Associated injury	Initial treatment	Re-operation
1	Male	36	Left	Fall	None	Plaster external fixation PRUJ and DRUJ	
2	Male	35	Right	Fall	Fracture ulnar coronoid process,	Hinged external fixation PRUJ	
					radial head	Repair LCL, MCL and capsule	
3	Male	20	Right	Traffic accident	Fracture ulnar coronoid process	ORIF ulnar coronoid process	
						Repair LCL and capsule	
4	Male	18	Left	Fall	Fracture ulnar coronoid process	Repair LCL	Release elbow contracture
5	Female	30	Left	Traffic accident	Fracture radial shaft	ORIF radial shaft	
6	Male	51	Right	Fall	Fracture radial shaft	ORIF radial shaft	
7	Male	44	Right	Fall	Fracture radial shaft	ORIF radial shaft	
						Hinged external fixation PRUJ	
8	Male	36	Right	Fall	Fracture radial shaft	ORIF radial shaft	
9	Male	29	Right	Machine accident	Fracture humeral/ulnar/radial Shaft, ulnar styloid process	ORIF humeral/ulnar/radial shaft	ORIF radial shaft
						K wire humeroradial joint	Neurolysis radial nerve
					Injury radial nerve		
					Subluxation DRUJ		
10	Female	46	Left	Traffic accident	Open fracture distal ulna/radius, ulnar styloid process	ORIF humeral shaft, distal ulna/radius, ulnar styloid process	
					Fracture humeral shaft, rib		
					Dislocation DRUJ Traumatic pneumothorax		
11	Male	18	Right	Fall	Fracture distal radius	Plaster external fixation PRUJ and DRUJ	
					Dislocation DRUJ		
12	Female	33	Left	Machine accident	Open fracture distal ulna/radius, ulnar styloid process	ORIF distal ulna/radius	
						Repair LCL	
No	Sex	Age	Side	Causes of injury	Associated injury	Initial treatment	Re-operation
					Fracture ulnar coronoid process		
					Dislocation DRUJ		
13	Male	28	Left	Fall	Fracture distal ulna/radius, ulnar styloid process	ORIF distal ulna/radius	
						Hinged external fixation PRUJ	
					Dislocation DRUJ	Repair LCL, MCL	
14	Male	17	Right	Machine accident	Fracture distal radius, ulnar styloid process	ORIF distal radius	
					Dislocation DRUJ		

*PRUJ*, proximal radioulnar joint; *DRUJ*, distal radioulnar joint; *LCL*, lateral collateral ligament; *MCL*, medial collateral ligament; *ORIF*, open reduction and internal fixation; *K wire*, Kirschner wire

**Fig. 3:**
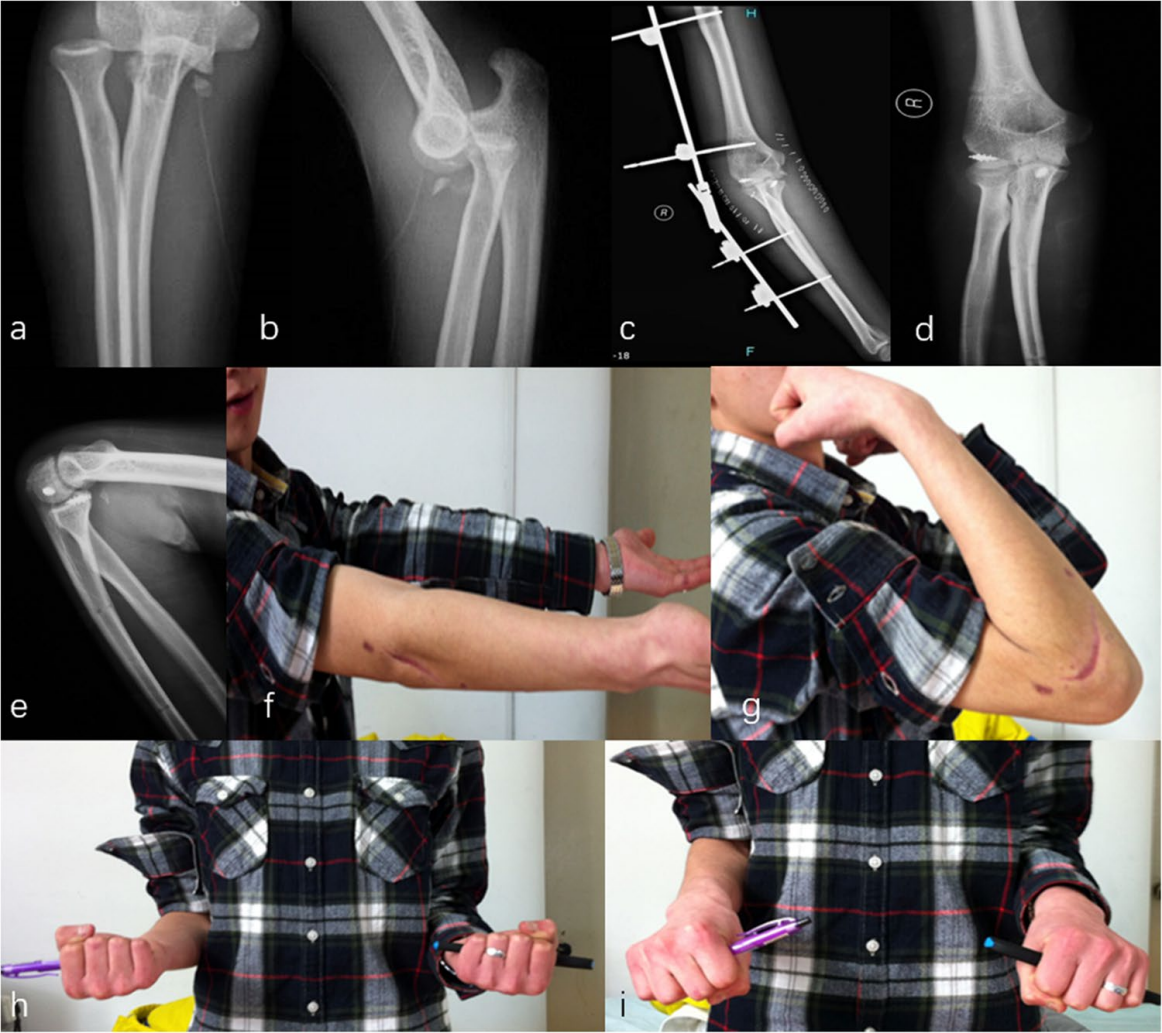
A case of a 35-year-old right-hand dominant male sustained a DDE combined with an ulnar coronoid process fracture (**a**–**b**). The patient was treated with an external fixator and a repair of the elbow joint ligament. The relatively tiny fracture fragments were not specifically addressed (**c**–**e**). Worth mentioning was the full range of motion was obtained at 12 monthly post-operative (**f**–**i**)

**Fig. 4:**
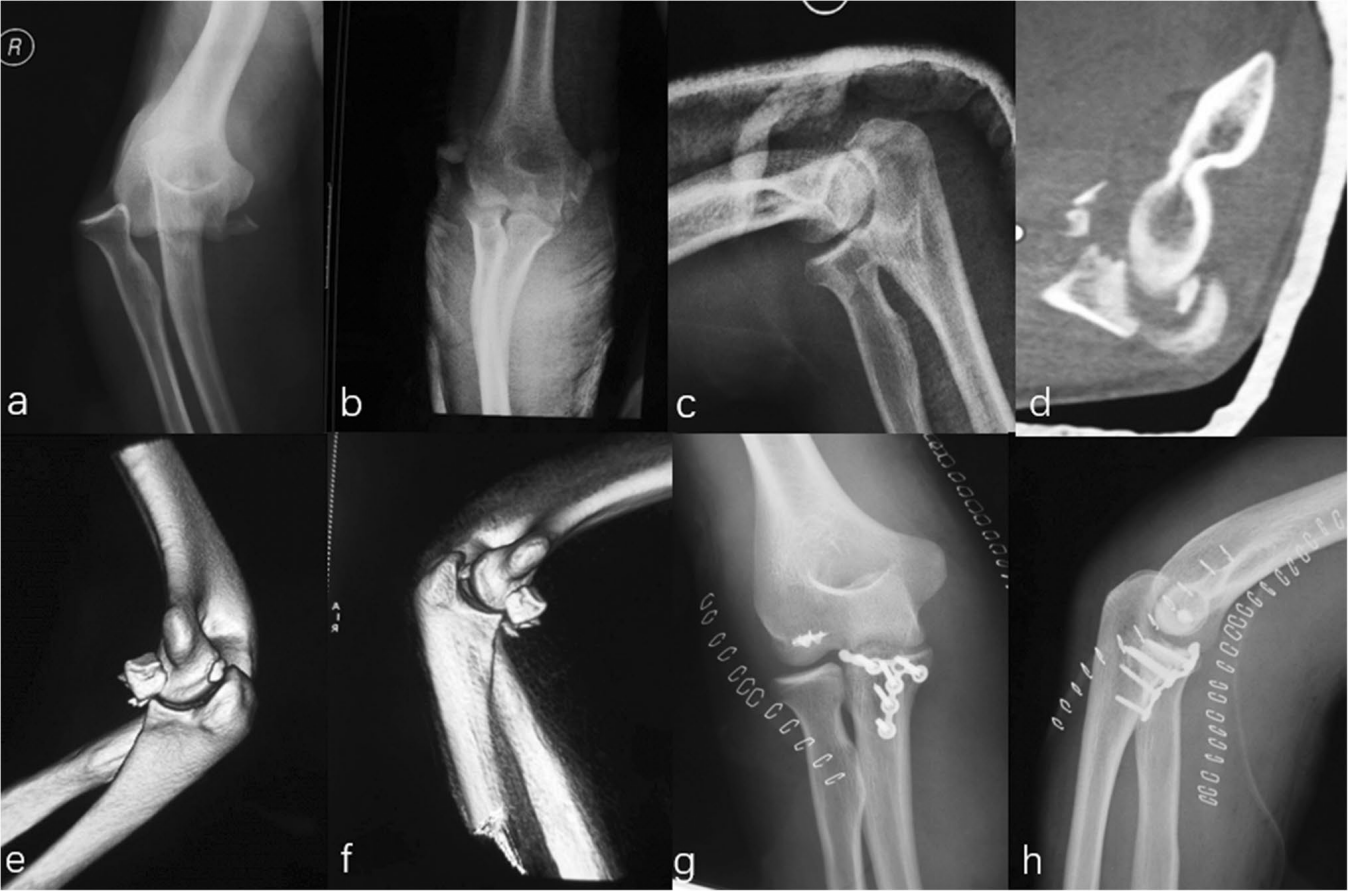
A 20-year-old male suffering a DDE and ulnar coronoid process fracture. Closed reduction was performed immediately (**a**–**b**). However, post-operative radiographs presented the ulnar coronoid process fracture was visibly large and lodged in the elbow joint (**c**–**f**). In addition, ORIF of the ulnar coronoid process and a repair of the LCL were also applied (**g**–**h**)

**Fig. 5:**
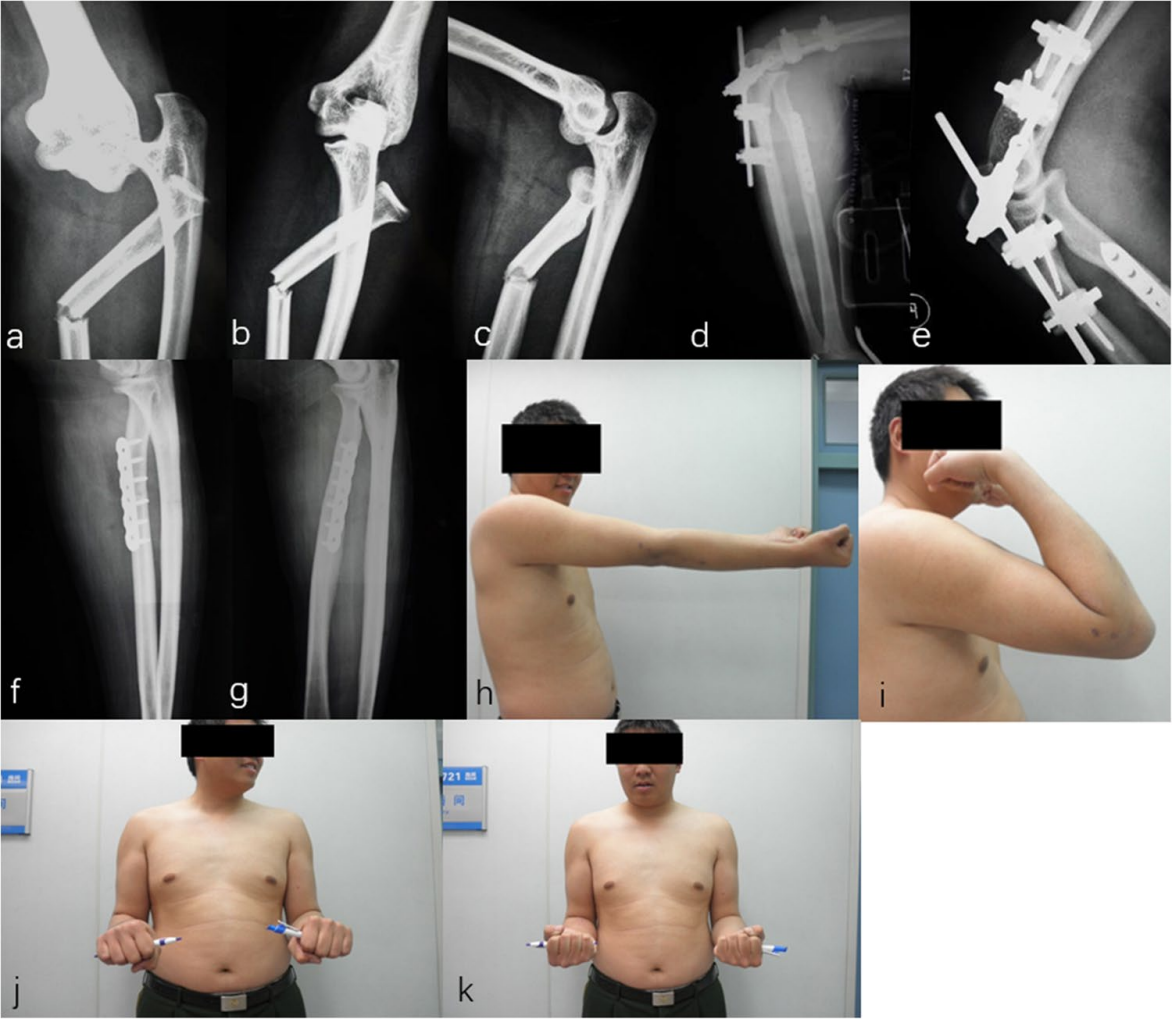
Radiographs of a 44-year-old right-hand dominant male exhibiting a DDE and a radial shaft fracture (**a**–**c**). Post-operative radiographs of the elbow showed good reduction and fixation with an external fixator and an ORIF of the radial shaft (**d**–**e**). The external fixator was removed at 6 weeks post-operative (**f**–**g**) and he has full range of motion at 18 monthly follow-up (**h**–**k**)

Patients that combined with unstable elbow fractures required surgical treatment. For the concomitant elbow periarticular fracture, surgeons should double-check the stability of elbow joints during operation. Especially when there was an apparent rupture of the joint capsule with complete tears of the lateral and medial collateral ligaments. The origins of the lateral collateral ligament (LCL) were reattached to the lateral epicondyle with suture anchors (Arthrex, Florida, USA) through the lateral Kocher approach [30]. The medial “over the top” approach was applied to stabilize the type II ulnar coronoid process fracture (according to the Regan and Morrey classification) with a precontoured plate (Accumed, Portland, Oregon) [31, 32]. Surgeons also preferred repairing the anterior oblique ligament of the medial lateral ligament (MCL) and anterior joint capsule with suture anchors (Figs. 3 and 4). The relatively tiny fracture fragments of the radial head and coronoid process were not explicitly addressed. After confirming that the proximal radioulnar joint (PRUJ) was corrected and the instability of the radial head, one humeroradial joint was fixed with a 2.5-mm Kirschner wire (K wire) in 60° flexion of the elbow and the neutral position of the forearm. Of note, cross or parallel K wires fixation of the PRUJ should be avoided in this fresh injury, which seriously limited early rehabilitation (particularly in the extension of the elbow). Furthermore, Fig. 5 demonstrates that one patient was applicated with an additional hinged elbow external fixator (Stryker Corp., Kalamazoo, MI, USA).

Two patients with open fracture-dislocation required staged operation. Firstly, sufficient irrigation and debridement were performed. The fracture-dislocation was provisionally reduced with K wires and a splint. Several days later, open reduction and internal fixation (ORIF) was performed for combined fractures (humeral/ulnar/radial shaft and the ulnar styloid process) with locking compression plates (LCP) or hook plates (Synthes, Paoli, USA).

**Fig. 6:**
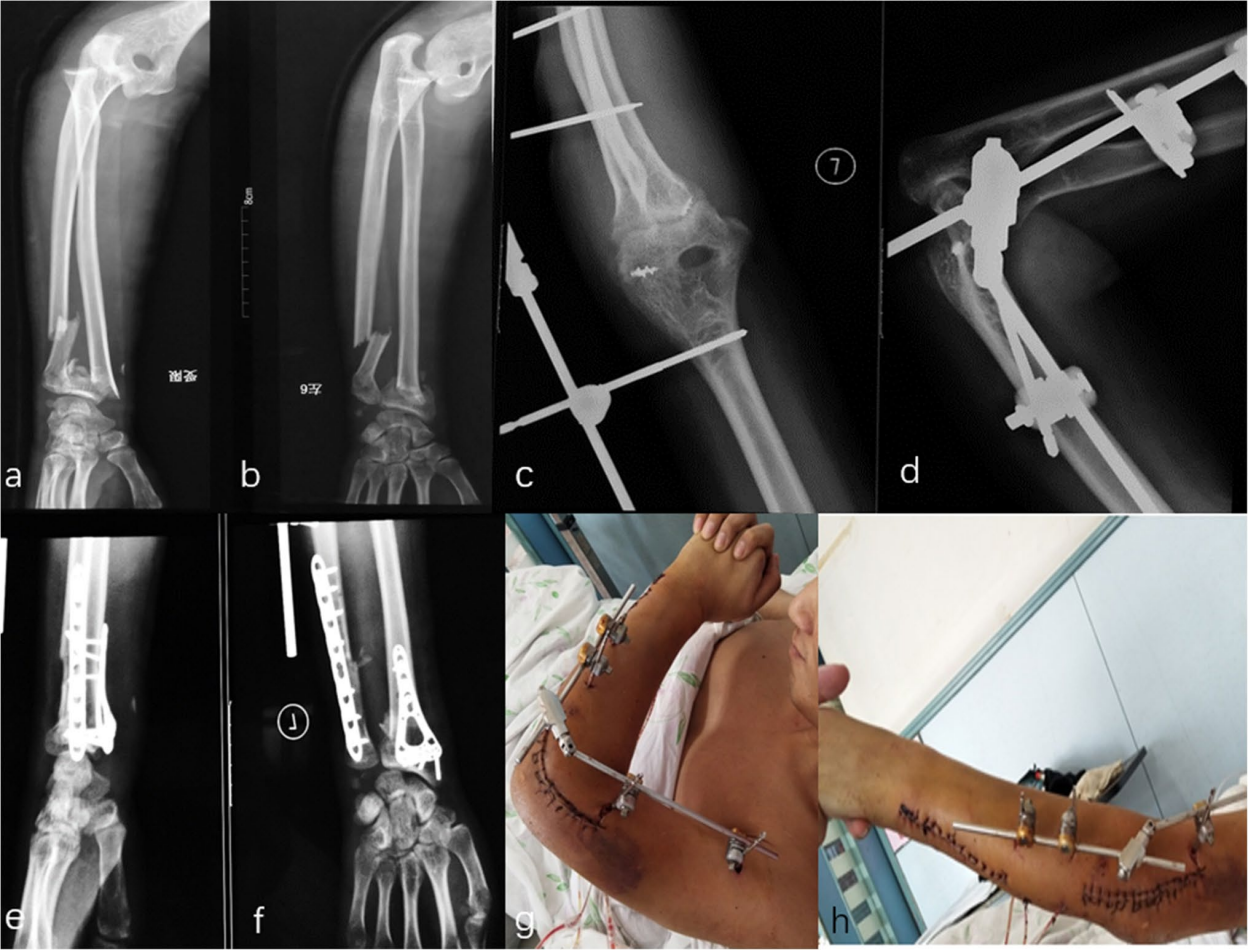
Anteroposterior and lateral radiographs revealed a DDE combined with distal ulna fracture, distal radius fracture and dislocation of DRUJ (**a**–**b**). Radiographic and clinical evidence demonstrated maintenance of the reduction of the PRUJ and DRUJ (**c**–**h**)

In addition, it was worth mentioning that the distal radioulnar joint (DRUJ) was stable after fracture reduction and fixation (Fig. 6).

### Post-operative treatment

The post-operative treatment depended on the severity of fracture-dislocations and clinical outcomes. Patients without hinged external fixators or pin-crossings were splinted in 90° of flexion for two to three weeks with the forearm in the neutral position. Active mobilization, including forearm rotation and elbow flexion and extension, was encouraged after two weeks under the guidance of doctors. Range of motion was gradually increased at three weeks postoperatively.

Active and passive elbow mobilization within pain limits started immediately for the patients with hinged external fixators. The fixator was not removed until six weeks post-operatively, and rehabilitation was continued without restriction. The DRUJ and humeroradial K wires were removed in four weeks.

Resistance exercises were performed when bone healing was obtained.

In addition, non-steroid anti-inflammatories (Indomethacin 25 mg orally three times daily) were administered for three weeks to prevent heterotopic ossifications.

### Clinical evaluation

There were 14 patients contacted and returned for at least 18 months of follow-up, including clinical outcomes, radiographic evaluation, and measurement of the range of motion using a goniometer. Follow-up evaluation was undertaken by the same surgeon who performed the operation.

The function of the wrist was assessed using the Mayo Modified Wrist Score (MMWS) [33]. The elbow function was assessed using the Mayo Elbow Performance Score (MEPS) and the Broberg and Morrey rating index [34, 35]. The Disabilities of the Arm, Shoulder and Hand (DASH) score was also performed [36]. Valgus and varus activity was tested in maximum extension and in 30° of flexion. The pivot-shift test was performed to evaluate posterolateral rotation, graded as normal, mild, moderate, or severely unstable [37]. Pain at rest and during the activity was assessed using a visual analogue score (VAS, 0 to 10, no pain to severe pain).

The radiographic examination was performed for fracture union, congruency of the humeroulnar and humeroradial joints, and signs of degenerative arthritis. Degeneration was classified according to Broberg and Morrey as grade 0 (normal joint), grade 1 (slight joint space narrowing and minimum osteophyte formation), grade 2 (moderate joint space narrowing and osteophyte formation), or grade 3 (severe degenerative changes with gross destruction of the joint). Fracture union was referred to bridging bone on anteroposterior and lateral radiographs. Heterotopic ossification was graded according to Brooker as grade I, II, III, or IV [38].

### Statistical analysis

The software SPSS 24.0 (IBM Corp. Armonk, NY, USA) was used for the statistical analysis of all follow-up data. Continuous variables were summarized as means (ranges).

## Results

Twenty-two adult patients were collected in the institutional database to be diagnosed with DDE between September 2010 and 31 March 2020. Of these, eight patients were excluded: four had a follow-up time of less than 12 months and four received a secondary procedure after failed initial treatment in another hospital. Finally, there were fourteen patients available for clinical and radiological review for an average of 53.2 months, with 11 males and two females, ranging from 17 to 51 years (the average age was 31.5 years) (Table 1). These patients’ left and right sides were injured in six and eight cases. The mechanism of injury involved falling from a great height (8 cases), traffic accidents (3 cases), and machine accidents (3 cases).

Of these fourteen patients, one patient was an isolated DDE. Three patients sustained combined ulnar coronoid process fractures, and one of them with a radial head fracture. Four patients underwent simultaneous radial shaft fractures. The remaining six patients presented the bipolar fracture-dislocation of the forearm (DDE + DRUJ subluxation/ dislocation + at least one fracture of the ulnar and radius). According to Gustilo and Anderson classification, there were two patients with open wounds at the forearm classified as type II and type IIIA, respectively [39, 40]. One patient suffered an ipsilateral radial nerve injury and one had multiple injuries with traumatic pneumothorax and rib fractures in the accident.

The mean activity of the elbow was from 133.2° (100 to 140°) of flexion to 8° (0 to 20°) of extension (Table 2). The average range of motion of the wrist was from 78.2° (60 to 90°) of flexion to 74.2° (40 to 90°) of extension. No patients showed elbow or wrist instability. The mean grip strength was 90.1% (81 to 99%), compared with the uninjured side. The mean pronation and supination of the forearm were 81.1° (10 to 90°) and 82.2° (45 to 90°), respectively. Functional scores were performed at the final follow-ups (Table 3). Of these 14 patients, the mean Mayo Modified Wrist Score (MMWS) was 91.4 points (80 to 100) (8 excellent (57%), six good (43%)), and the mean Mayo Elbow Performance Score (MEPS) was 93.4 points (70 to 100) (7 excellent (50%), five good (35.7%), two fair (14.3%)). The mean Broberg and Morrey functional index was 92.6 points (79 to 100) (7 excellent (50%), five good (35.7%), two fair (14.3%)). The mean Disabilities of the Arm, Shoulder and Hand (DASH) score was 10.7 (0 to 36). In addition, minimal pain was reported with a mean Visual Analogue Scale (VAS) score of 0.4 (0 to 1) at rest and 1.7 (0 to 3) during activities.

**Table 2:** Post-operative results (range of motion)

Number	Follow-ups	Elbow		Wrist			Forearm	
		Flexion (°)	Extension (°)	Flexion (°)	Extension (°)	Grip (%)	Pronation (°)	Supination (°)
1	50	138	13	85	80	90	89	88
2	35	139	11	82	81	92	88	87
3	110	135	10	90	90	96	87	90
4	53	136	11	78	72	91	68	45
5	18	138	6	80	77	93	88	86
6	56	139	4	74	83	90	87	85
7	52	140	20	88	86	99	86	87
8	38	132	17	87	85	94	86	84
9	59	100	11	60	40	88	90	86
10	46	121	0	68	48	81	10	72
11	76	134	4	70	70	91	88	88
12	65	136	2	78	75	84	89	86
13	45	139	2	76	80	87	90	85
14	42	138	1	79	72	86	90	82

**Table 3:** Evaluation scores of clinical outcomes

Number	MMWS	MEPS	Broberg-Morrey functional index	DASH score	VAS	
					At rest	During activities
1	E	E	E	4	0	1
2	E	E	G	6	0	2
3	E	E	E	8	0	2
4	G	G	F	13	0	2
5	E	E	E	7	1	2
6	E	E	E	9	0	2
7	E	G	E	11	0	2
8	E	E	E	0	0	0
9	G	F	F	36	1	3
10	G	G	G	16	0	2
11	E	E	E	9	1	1
12	G	G	G	11	0	2
13	G	G	G	8	1	2
14	G	F	G	12	1	1

*MMWS*, Mayo Modified Wrist Score; *MEPS*, Mayo Elbow Performance Score; *DASH*, Disabilities of the Arm; Shoulder or Hand; *VAS*, Visual Analogue Scale; *E*, excellent; *G*, good; *F*, fair

**Fig. 7:**
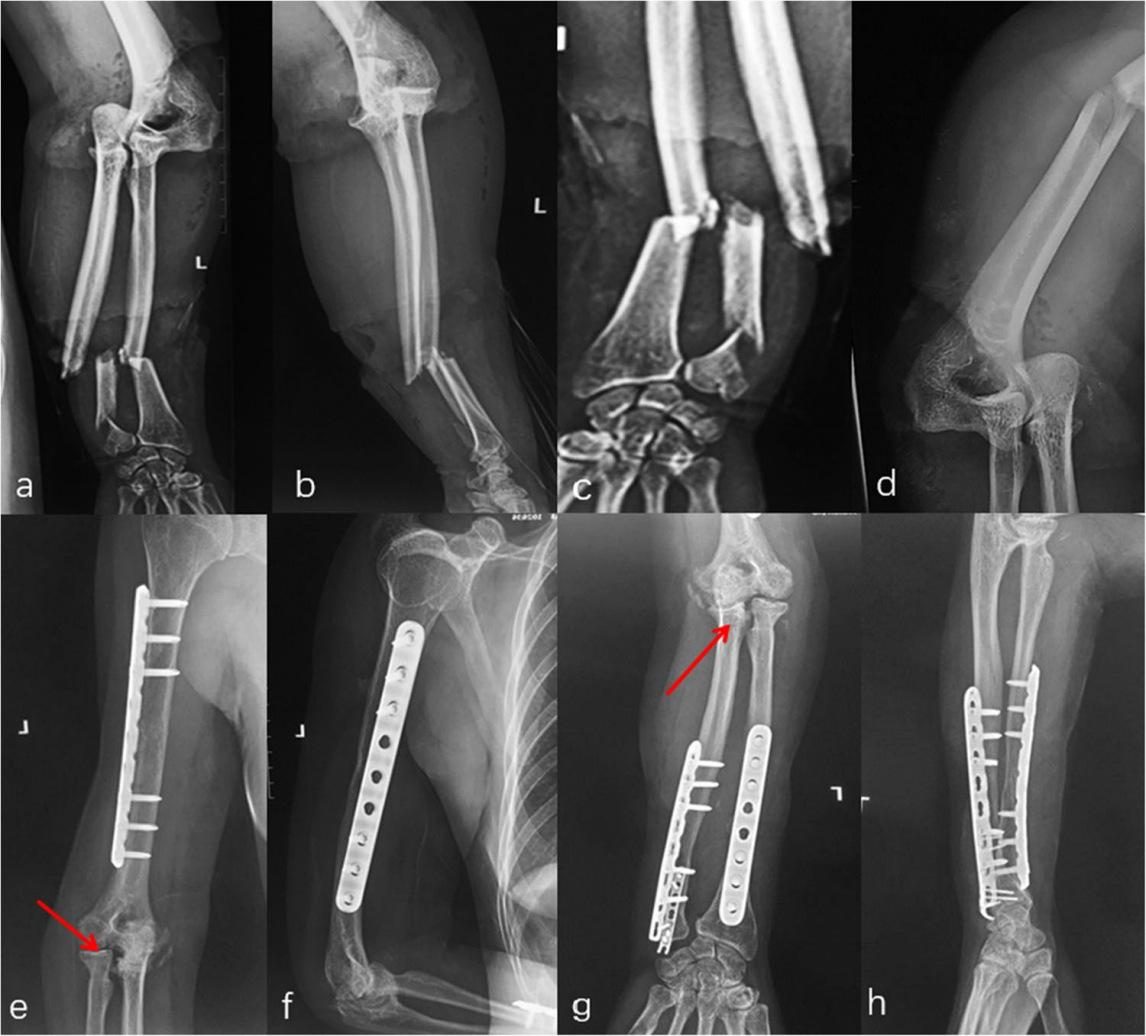
Initial radiographs of the elbow and wrist showed the DDE and a multiple fracture (**a**–**d**). All fractures were reduced by internal fixation and subsequent X-ray revealed a grade 1 heterotopic ossification at a 12 monthly follow-up (arrow) (**e**–**h**)

**Fig. 8:**
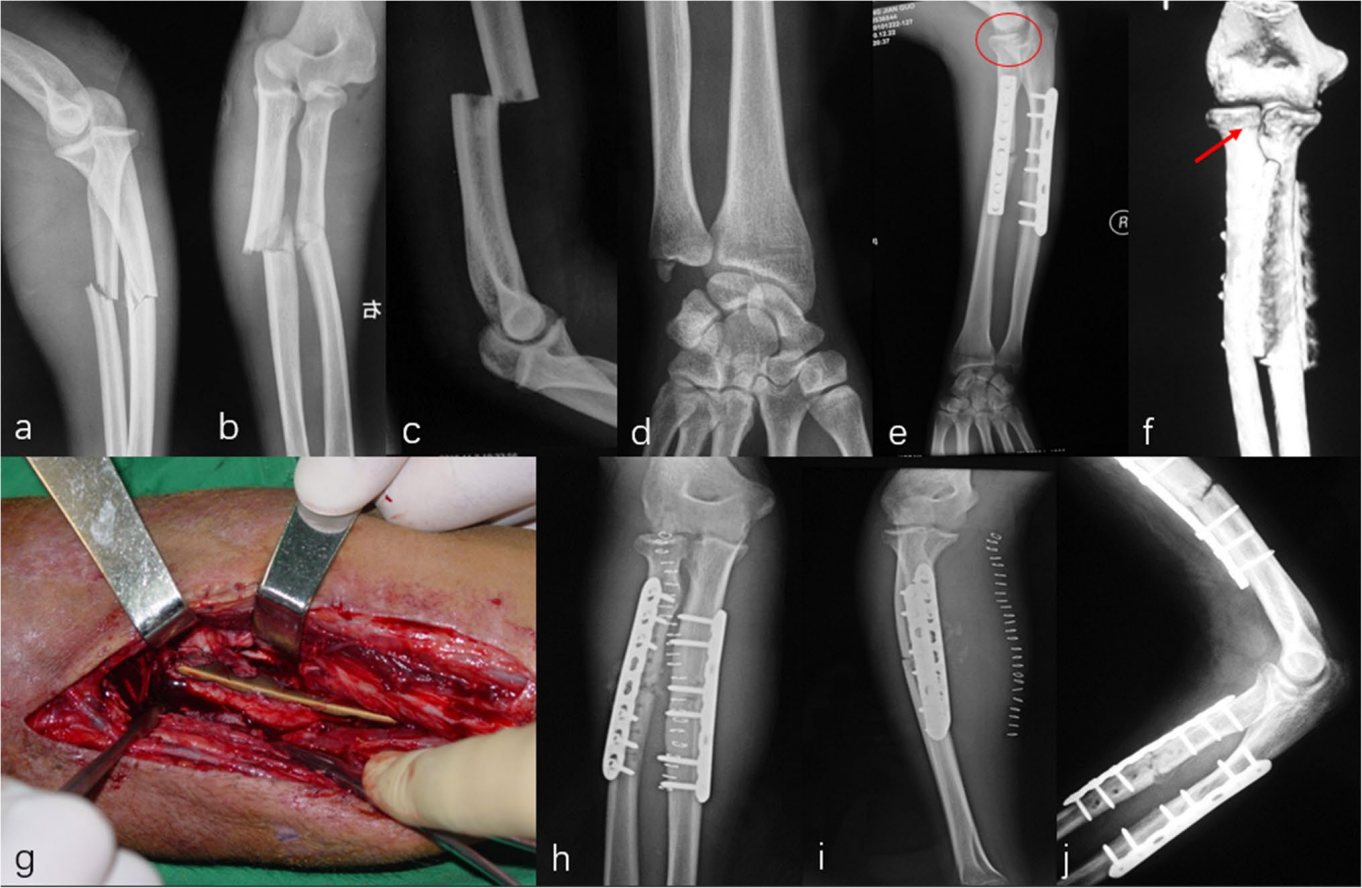
Post-operative radiographs of the right elbow revealed a DDE and a multiple fracture (**a**–**d**). However, this patient sustained an elbow malrotation (arrow and circle), which failed to restore the anatomical radial bow (**e**–**f**). Eight weeks later, a secondary operation consisting of radius shortening and release of an elbow contracture was required to restore motion (**g**–**j**)

**Fig. 9:**
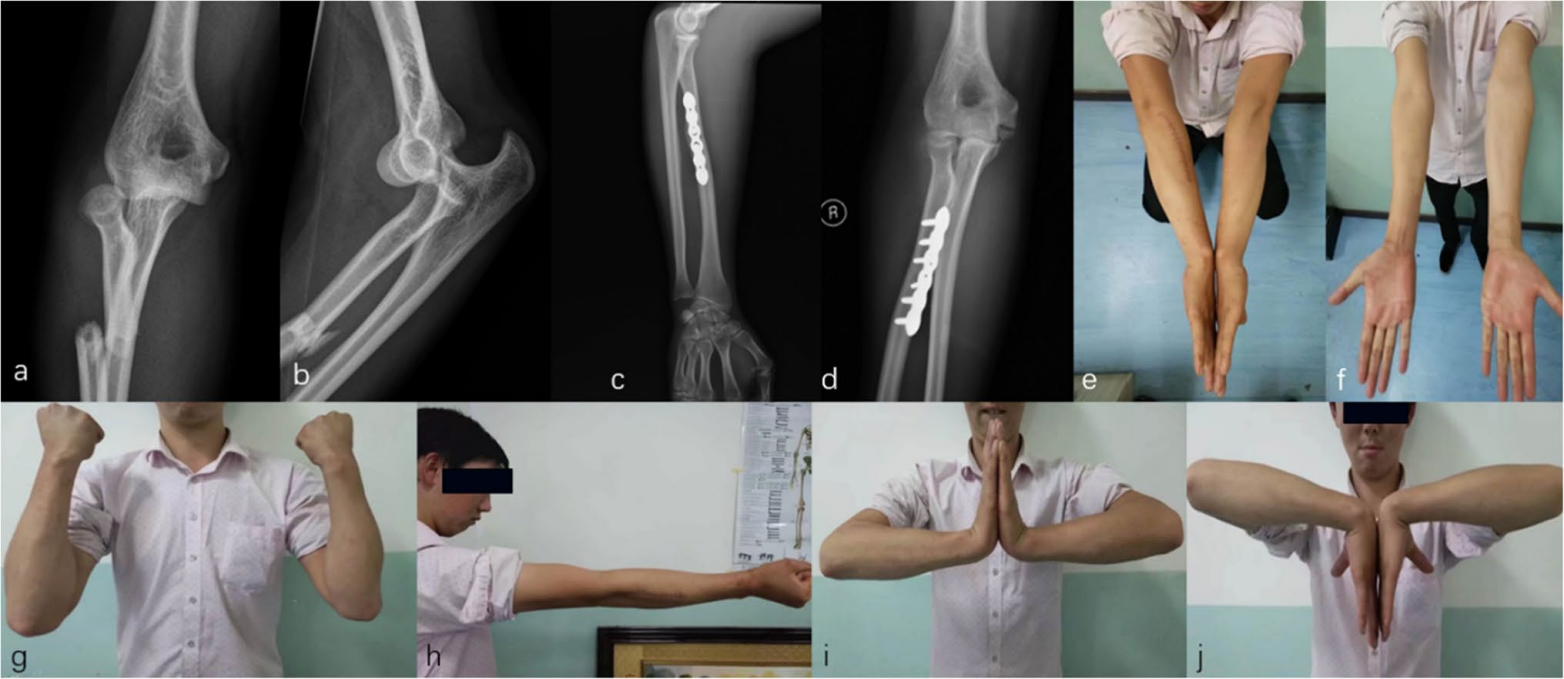
**a**–**b** One case suffering a DDE combined with a radial shaft fracture: he received surgical treatment immediately and was found asymptomatic grade 1 heterotopic ossification (**c**–**d**). Clinical photos of the elbow 2 years after surgery showed an excellent clinical outcome compared with the opposite side after a regular rehabilitation training (**e**–**j**)

Eleven patients were found great radiographic outcomes without any sign of degeneration or heterotopic ossification in the elbow region. Two patients, combined with radius/ulna shaft fracture and DRUJ injury, showed grade 1 degeneration (Figs. 7 and 8). Figures 7 and 9 also involve an asymptomatic grade 1 heterotopic ossification.

Of note, case 10 presented a slight hypoaesthesia of the radial nerve on admission, which was attributed to the fracture displacement and traction of the nerve. Therefore, surgeons performed the ORIF without radial nerve exploration. At three months follow-up, the patient demonstrated that the forearm muscle strength was recovered and the Tinel test presented the dorsoradial paralysis disappeared. Finally, this symptom resolved entirely at six months postinjury.

Two patients received secondary treatments. One case was attributed to elbow malrotation, which failed to restore the anatomical radial bow inhibiting the humeroulnar joint reduction (Fig. 8). The revision surgery was performed at eight weeks after the initial operation, consisting of malalignment correction, re-fixation with a 3.5 mm locking compression plate, and iliac bone autograft for the radial shaft fracture and the elbow reduction. The clinical outcome was satisfactory compared with the opposite side. The other one underwent subsequent surgery for elbow contracture release, with an activity of flexion and extension was 70° and 5° for the elbow. Then, the range of motion improved to 130° and 5° after secondary treatment.

## Discussion

Although studies regarding pediatric cases have been increasingly published, literature reporting DDE in adults remained sparse. In 1854, Warmont first described this trauma in a child [41]. In 1981, DeLee reported the first case of divergent elbow dislocation confirmed by radiographic evidence [1]. We collected the most extensive series of adult patients with clearly defined traumatic DDE and the clinical outcomes were also assessed. In contrast to paediatric cases that DDE is mainly caused by a simple fall, high-energy trauma frequently presents in adults, which results in associated fracture/dislocation of the forearm. Our study divided patients into four types of injury patterns according to the combined fractures of forearms as follows:

Type A isolated DDE (1/14, 7.1%).

Type B DDE + periarticular elbow fractures (radial head fracture, ulnar coronoid fracture, etc.) (3/14, 21.4%).

Type C DDE + radial and/or ulnar shaft fracture (4/14, 28.6%).

Type D bipolar fracture-dislocation of the forearm (DDE + DRUJ subluxation/ dislocation + at least one ulnar and radius fracture) (6/14, 42.9%).

Each type in our study was demonstrated in previous literature [2–6]. These concomitant fracture-dislocations of the forearms further complicated the DDE in adults, which brought a considerable challenge to orthopaedic surgeons.

In a recent study, DDE was associated with periarticular elbow disruptions, including the LCL, MCL, interosseous membrane, and capsule. This injury consisted of anteroposterior and mediolateral (or transverse) dislocations. However, our study found that the anteroposterior and lateral views of the elbow joint showed the radial head was dislocated laterally and the proximal ulna was dislocated in a posteromedial direction, respectively. A nonstandard radiographic analysis always implied a confounding diagnosis of two types of DDE. Hence, our results were consistent with Altuntas et al., which demonstrated that only the posterior DDE existed [17]. With regard to the significance of radial head position and other traumas (forearm and distal radial fractures, interosseous membrane and DRUJ instability, etc.) in initial clinical examination, our study preferred a pre-operative CT scanning for patients with DDE of high-energy injury.

The operative indication for DDE has become into question because of its complex anatomical structure. Previous literature stated that a resultant and axial violence is applied parallel to the longitudinal axis of the forearm and in association with the rupture of the annular ligament and interosseous membrane [2, 12, 13]. Cadaver studies demonstrated that radial head dislocation occurred by pronating violence of the forearm after the MCL rupture existed. This evidence showed that the forearm was in a position of supination and the displaced humerus divorced the radius and ulna. Moreover, recent literature mentioned that annular ligament (AL) reconstruction should be considered due to a posterolateral dislocated radial head [6]. However, our surgeon did not find the AL damage due to the dislocated radial head during surgery.

For paediatric cases, physiologic or pathologic joint laxity related to three articulations separation is highly unusual. Casstevens et al. proposed that a valgus stress and axial loading due to an outstretched forearm with soft-tissue lesions was the primary injury mechanism for a traumatic DDE [3]. Recently, Greene et al. presented a traumatic DDE case receiving a forearm amputation. This grown patient underwent a machine accident caused by auger type, who was observed with generalized swelling of the elbow, severe torsion, ulnar and radial shaft fractures, an LCL tear, and traumatic amputation of the distal aspect of the forearm and the wrist [5]. A case report revealed that a patient with Ehlers-Danlos syndrome (EDS) fell while snowboarding and developed a DDE without a fracture that required an ORIF [6]. Clinical features of this disease included soft-tissue fragility, hyperextension, and joint laxity. However, the exact traumatic mechanism remained unknown, and at least one above-mentioned factor was involved in the present cases.

Closed reduction for DDE in most pediatric cases could obtain a great eventual outcome if the diagnosis was precise after admission [23]. Reduction techniques have been mentioned in published studies [1–3, 7, 8, 11, 13]. For patients with a persistent unstable PRUJ/ DRUJ, an additional hinged external fixator or K wire fixation was optional. Considering the difference in the severe nature of the injury between DDE in adults and children, surgeons should pay particular attention to ORIF combined ligamentous reconstructions in high-energy cases, which provide patients with great possibilities of maintaining long-term painless rehabilitation without complications. In our study, the overall function outcome was satisfactory, consistent with the previous works in adults (Table 4).

**Table 4:** Cases of traumatic DDE in adult patients previously reported in the literature

No	First author	Year	Age	Sex	Causes of injury	Associated fractures	Treatment
1	Kazuki^2^	2005	41	Male	Fall	Fracture humeral shaft, radial head, ulnar coronoid and styloid process and distal radius	OR
2	Casstevens^3^	2012	29	Male	Fall	Fracture humeral shaft, scaphoid, trapezium and fourth and fifth metacarpal	OR
3	Laratta^4^	2014	47	Female	Fall	Fracture radial head, ulnar coronoid process and radial shaft	OR
4	Greene^5^	2018	49	Male	Machine accident	Fracture ulnar coronoid process and shaft	OR
5	Onode^6^	2021	32	Male	Fall	Fracture ulnar coronoid process	OR

*DDE*, divergent dislocation of the elbow; *OR*, open reduction

Until now, it remains an ongoing debate for standard treatment of DDE in adults due to the infrequency and diversity of this damage and the relatively insufficient data available in the previous literature. Nonetheless, according to our experience and the clinical data, the following principles are presented: (i) The elbow dislocation should be reduced immediately. Clinical tests and radiographic evidence are conducted to check the residual elbow instability. (ii) The conservative treatment with closed reduction is an alternative option for the isolated DDE, providing sufficient humeroulnar and PRUJ stability. (iii) DDE combined with elbow periarticular fractures (such as the ulnar coronoid process fracture, radial head/neck fracture) or the ligaments/capsule disruption should be treated with ORIF because of its unstable structure and all three elbow articulations involved, which requires anatomic reduction and stable plate fixation to restore the concentric elbow stability and permits early mobilization. It would be advisable that the forearm fracture acquires anatomic alignment, contributing to DDE reduction. Occasionally, the DDE combined with a stable fracture received a closed reduction and was maintained with a splint (Table 1, no. 11). (iv) The elbow joint stability is checked in a full range of motion. Any residual instability should be treated with additional LCL /MCL repair. (v) We suggest paying particular attention to the DRUJ and the forearm longitudinal stable. Currently, four complex DDE combined with the DRUJ instability are referred to Essex-Lopresti injuries. In this situation, three lockers of the forearm ring are destroyed, causing longitudinal instability of the forearm. Then, the elbow joint, PRUJ and DRUJ stability should be confirmed and further repair to prevent residual instability. (vi) Early post-operative functional training is recommended, and elbow immobilization should not exceed three weeks to avoid stiffness. When residual post-operative instability or the related reconstruction is found to be tenuous, a hinged elbow fixation results in an excellent clinical outcome.

This study has several limitations worth mentioning, including its retrospective design. The relatively small series size limits the reliability of our study. However, our study included the largest number of cases in DDE-related literature. There is no consensus on the treatment principle of DDE, causing a lack of consistent treatment algorithms. Of note, this study does not consist of any case with delayed treatment (more than 2 weeks after injury) due to its more complex pathogenic condition and surgical treatment. In addition, several surgical interventions caused a longitudinal separation of the forearm and poor clinical outcomes. Our institution will report a series of delayed treatments for elbow dislocation in future studies.

## Conclusion

Based on providing the anatomic stability of the elbow joints, the treatment effect for DDE with concomitant injuries could be relatively warranted. In DDE associated with periarticular fractures (and/or ligamentous injuries), the closed reduction and subsequent procedures, including ORIF (and/or ligament repair) and early rehabilitation training, are performed instantly. In addition, early rehabilitation training under the guidance of doctors was beneficial for eventual outcomes.

## Data Availability

The data and material that support the findings of this study are available from the corresponding author upon reasonable request.
